# Exercise Reduces Competition between Procedural and Declarative Memory Systems

**DOI:** 10.1523/ENEURO.0070-20.2020

**Published:** 2020-07-21

**Authors:** Jing Chen, Marc Roig, David L. Wright

**Affiliations:** 1 Department of Kinesiology, Texas A&M University-Texarkana, Texarkana, TX 75503; 2 Memory and Motor Rehabilitation Laboratory (MEMORY-LAB), Feil and Oberfeld Research Centre, Jewish Rehabilitation Hospital, Montreal Center for Interdisciplinary Research in Rehabilitation (CRIR), Laval, Quebec H7V 1R2, Canada; 3 School of Physical and Occupational Therapy, Faculty of Medicine, McGill University, Montreal, Quebec H3A 0G4, Canada; 4 Non-Invasive Brain Stimulation Laboratory, Department of Kinesiology, Texas A&M University, College Station, TX 77840

**Keywords:** consolidation, declarative learning, exercise, motor sequence skill, procedural skill

## Abstract

The neural systems that govern declarative and procedural memory processing do not always operate independently. Direct evidence of competition between these two memory systems in humans is supported by studies showing that performing a declarative learning task immediately after motor skill learning can disrupt procedural memory and abolish the off-line gains in skill performance obtained during consolidation. The aim of the present study was to extend recent investigations demonstrating that the exposure to a brief bout of cardiovascular exercise can protect procedural memory by enhancing postpractice consolidation. We used an experimental paradigm designed to assess whether exercise can also protect procedural memory consolidation from interference induced with declarative learning. The implicit acquisition of a serial reaction time task (SRTT) was tested after a 6-h waked-filled period. Participants who were exposed to a non-learning vowel counting (VC) task following the practice of the SRTT exhibited successful procedural memory consolidation and significant off-line gains in skill performance. Confirming that declarative memory processes can interfere with procedural memory consolidation, off-line gains in motor skill performance were suppressed when the performance of the VC task was replaced with a word list (WL) task requiring declarative learning. Performing a bout of cardiovascular exercise after the SRTT protected the newly formed procedural memory from the interference produced by the WL task. Protection was evidenced by a return of significant off-line gains in skill performance after the waked-filled period. Exercise optimizes the utilization of neural resources reducing interference between procedural and declarative memory systems.

## Significance Statement

Our memory system is extremely selective and imposes a “bottleneck” limiting the capacity to process multiple memories simultaneously. Competition between memories occurs between memories of the same memory system and between memories relying on different memory systems. This study demonstrates that cardiovascular exercise can protect a procedural memory from interference induced by declarative learning. We show that a bout of exercise between the practice of a motor skill acquired implicitly and declarative learning protects the newly formed procedural memory from declarative interference, promoting off-line gains in skill performance after a period without sleep. Our findings suggest that exercise optimizes the use of neural resources during the simultaneous processing of memories by reducing competition between memory systems.

## Introduction

Cardiovascular exercise can be used as a simple and affordable intervention for improving different types of learning and memory (Roig et al., 2013). For procedural learning, a robust long-term benefit is associated with just a single session of exercise (Roig et al., 2012). When performed shortly after practice, cardiovascular exercise facilitates procedural learning by improving memory consolidation (Roig et al., 2016). As such, several investigations have demonstrated that a single bout of exercise performed in close temporal proximity to motor skill practice enhances the long-term retention of the motor skill (Roig et al., 2012; Mang et al., 2014; Skriver et al., 2014; Thomas et al., 2016a,b). Depending on the motor task used, exercise-induced enhancements in procedural memory during consolidation manifest either as a preservation of the motor skill (i.e., stabilization) or as an increase in skill performance despite no additional motor practice (i.e., off-line gain; Robertson et al., 2004). Such enhancements in procedural memory consolidation occur after a period of sleep (Roig et al., 2012; Mang et al., 2014; Rhee et al., 2016; Thomas et al., 2016a,b,c) and after a wake period (Ostadan et al., 2016; Stavrinos and Coxon, 2017).

Acute cardiovascular exercise can also protect procedural memory from behavioral-induced interference during consolidation. Rhee et al. (2016) inserted 20 min of vigorous cardiovascular exercise (Garber et al., 2011) between the practice of a target motor sequence and additional practice with a novel interfering motor sequence performed 2 h later. Despite exposure to interference, a small off-line improvement was observed 24 h later for individuals who exercised compared with those who did not. Jo et al. (2019) expanded this work using a 6-h retention interval that did not include sleep and found similar protection for a novel procedural memory from interference through exercise (also see Lauber et al., 2017). However, all these studies used primary and secondary (i.e., interfering) motor tasks with overlapping internal models that competed for the same neural resources during memory processing (Zach et al., 2012). Whether acute exercise protects procedural memory from the interfering effects of tasks originating from other non-overlapping memory system (e.g., declarative system) is currently unknown.

Memory interference may be influenced by a competitive interaction that can occur between different memory systems (Albouy et al., 2008). For example, Brown and Robertson demonstrated that performing a declarative learning task immediately after motor skill learning can disrupt procedural memory and abolish off-line gains in skill performance during a period of consolidation without sleep (Brown and Robertson, 2007a). Identifying strategies to reduce interference between different memory systems is relevant because our brain is continuously challenged to process different types of memories seldom acquired in isolation. To evaluate whether exercise contributes to a brain state that optimizes the interaction between different memory systems, the present work attempted to replicate the interference from declarative learning on procedural consolidation (Brown and Robertson, 2007a) while also testing whether the inclusion of exercise could mitigate such interference. Specifically, we sought to determine the efficacy of an acute bout of cardiovascular exercise for facilitating procedural consolidation over a wake interval despite experiencing interference from supplemental declarative learning. A novel prediction was that exposure to cardiovascular exercise would nullify the interfering impact of declarative learning on procedural memory consolidation allowing off-line gains to occur across a 6-h wake-filled period.

Individuals learned an implicit version of a serial reaction time task (SRTT), which elicits off-line gains in motor skill performance after wake (Brown and Robertson, 2007a). One hour after the practice of the SRTT, participants performed either a declarative learning task designed to induce interference or a control vowel counting (VC) task, which did not require declarative learning. An additional experimental condition involved the insertion of a 20-min bout of cardiovascular exercise before the declarative learning task, which as in the previous two conditions, was also performed 1 h after the SRTT. The extent of procedural memory consolidation was inferred from the change in motor skill performance at the conclusion of training and the retention test administered after a wake-filled 6-h interval (Jo et al., 2019). It was expected that while an off-line gain for the SRTT would occur for individuals that experienced the control VC task, this gain would be abolished when a declarative learning word list (WL) task was included (Brown and Robertson, 2007a). Adding an acute bout of exercise after practice of the SRTT but before declarative learning was predicted to protect procedural memory, leading to off-line gains from consolidation across a wake interval to the level observed for the individuals exposed to the VC task.

## Materials and Methods

### Participants

A total of 112 undergraduate right-handed students with no neurologic or psychiatric condition and with no contraindications to exercise were recruited. All participants gave informed consent to take part in the experiments. At the end of the study, 40 participants were excluded from the analysis as a result of being able to recall, in a postexperiment verbal recall test (VRT), more than four elements in the 12-item sequence of the implicit version of the SRTT used in the study (see full description provided in following section, procedural learning). This requirement is applied to ensure that participants acquire this version of the SRTT implicitly, thus minimizing the involvement of declarative learning as much as possible (Willingham and Goedert-Eschmann, 1999). Satisfying this requirement was especially important in this study, where interference between procedural (i.e., non-declarative) and declarative memory processes was investigated. Importantly, the proportion of participants finally excluded (36%) was similar to what has been reported in previous studies (Brown and Robertson, 2007a). Data from 72 participants including 21 males and 51 females were included in the final analysis.

### Experimental design

**Figure 1.**
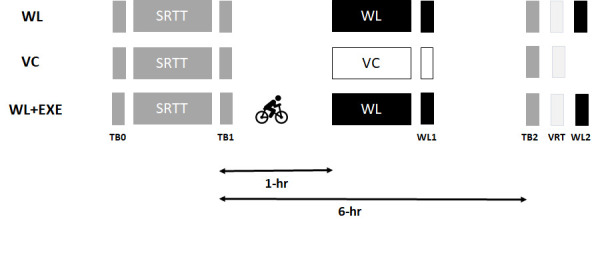
Three experimental conditions (WL, VC, and WL+EXE) were included. All participants practiced the SRTT (procedural skill), and skill was determined before (TB0) and at the conclusion of this bout of practice (TB1). Individuals in the WL condition then performed a WL recall task (declarative learning), which was subsequently tested 10 min after several repetition of the WL (WL1). A different set of individuals performed a vocal counting activity after practice of the SRTT (VC condition). This condition serves as a control. VC has been argued to engage the declarative system but does not involve learning. A final set of participants followed the same protocol as the WL condition with the addition of a bout of cardiovascular exercise immediately after practice with the SRTT but before exposure to the WL (WL+EXE condition). All participants completed an additional TB (TB2) with the SRTT 6 h after the initial training was completed as well as a VRT of the SRTT. For the individuals assigned to the WL and WL+EXE conditions, this was followed by a final WL recall (WL2).

The timeline for all phases of the experiment are provided in Figure 1. All participants were first exposed to motor practice with the SRTT. One hour after practice participants were randomly assigned to conditions that incorporated either a declarative learning activity that involved a WL task or an alternative non-learning control verbal task that involved VC. A third group of participants were assigned to the WL and exercise (WL+EXE) condition. In this condition, participants also performed the WL task 1 h after practicing the SRTT, but they experienced an acute bout of cardiovascular exercise immediately after motor practice was completed and before declarative learning. The purpose of introducing exercise at this point was to enhance the consolidation of procedural memory and determine whether exercise protects this memory from the interfering effects derived from declarative learning. Participants in all experimental conditions (WL, VC, WL+EXE) completed a test of the SRTT to assess motor skill retention 6 h after motor practice. Moreover, participants in conditions WL and WL+EXE completed a retention test of the WL task 6 h after having performed the declarative learning task.

### Procedural learning

A SRTT previously used to study implicit procedural learning was used (Robertson, 2007). Participants were comfortably seated in front of a computer screen. A solid circular visual cue appeared at any one of four possible positions organized horizontally in the lower third of the computer screen. The left most visual cue was labeled “1,” whereas the right most was labeled “4.” Each of the four horizontal positions corresponded to one of the four spatially compatible keys on a computer keypad on which the fingers of the participant’s right hand rested. When a circular cue was illuminated, participants were instructed to press the corresponding key on the keyboard as accurately and quickly as possible. The visual cue remained illuminated until the correct key was pressed. Having pressed the correct key, the cue on the screen disappeared and it was replaced by the next cue after a delay of 250 ms. The 12 visual cues for the SRTT presented on the computer screen followed the following order: 2–3–1–4–3–2–4–1–3–4–2–1. Importantly, the SRTT was explained to participants as a test of reaction time and, to minimize declarative knowledge of the sequence, they were not made aware of the existence of any repeating pattern.

Performance of the SRTT began with test block (TB)0, which involved 15 repetitions of the 12-item sequence (i.e., 180 trials). Data from TB0 was used to determine skill level at baseline. TB0 was followed by a longer period of practice that was made up of 25 repetitions (i.e., 300 trials). This period of practice was followed by TB1, which also included 15 repetitions of the repeated sequence (i.e., 180 trials). Data from TB1 was used to determine skill level post practice (skill 1). TB2 of the SRTT, that again consisted of a single block with 15 repetitions (i.e., 180 trials) of the repeated sequence, was performed 6 h after initial practice to assess skill retention (skill 2). Importantly, 50 random trials preceded and followed the blocks of trials with the repeating 12-item sequence. A VRT to assess each individuals’ explicit knowledge of the SRTT was performed after TB2. As previously stated, data of participants who reported knowledge of the correct ordinal position of more than four items in the 12-item sequence were removed from the analyses (Willingham and Goedert-Eschmann, 1999).

### Declarative learning

A WL task previously used and described as involving declarative learning was used (Brown and Robertson, 2007a,b). For this task, a word,selected from a predetermined set of 16 words drawn from the California verbal learning test, was presented on a computer monitor for 2 s. After the 2-s presentation of the initial word, a new word from the set was then presented. This presentation scheme continued until 16 words that constituted the learning list had been viewed by the participant. Once all 16 words had been viewed, participants were asked to recall, in any order, as many of the words from those just presented in the previous list. When this recall test was completed, the same 16 words were presented to the participant an additional four times, for a total of five presentations of the list, with the words being presented in the same order each time and recall being requested following each viewing of the complete set of 16 words. Ten minutes and 6 h after the fifth presentation of the WL, each individual was asked to complete immediate (WL1) and delayed free recall tests (WL2) of the WL, respectively.

### Vowel counting task (VC)

A VC task was used as the control condition for the declarative learning task. While this task engages the declarative system, it does not entail learning (Brown and Robertson, 2007a,b). Participants were shown a list of 16 nonsense letter strings, varying in length from three to 12 letters. The goal of the task was to count and then state the number of different vowels within a string. Each string was presented on the computer monitor for 2 s and, like the WL, involved a new letter string being presented until 16 nonsense letter strings had been viewed. Consistent with the protocol for the WL condition, each participant was exposed to five presentations of the list of 16 nonsense letter strings, completed the counting task, and articulated the vowel count after each trial. After the presentation of the fifth set was complete, a 10-min interval was allowed before the 16 nonsense letter strings were again presented followed by an assessment of the number of different vowels within each string. Any single nonsense letter string was not repeated.

### Exercise intervention

One experimental condition included in the experiment (WL+EXE) required participants to perform an acute bout of cardiovascular exercise between the practice of the SRTT (i.e., procedural learning) and the WL task (i.e., declarative learning; Fig. 1). Before any participation in the experiment, resting heart rate (RHR) was obtained from all participants using a HR monitor (Polar, EDC). To control for different fitness levels, the intensity of the acute exercise bout used during the experiment before completing the declarative learning activity was individually tailored using each participant’s HR reserve (HRR) calculated as:

$$\text{HRR}\;=\;({\text{HR}}_{\text{age}-\text{predicted max}}-\text{ RHR}),$$

where

${\text{HR}}_{\text{age}-\text{predictlineed max}}=\;208\;–\;\left(0.7\;\text{x age}\right)\;$(Tanaka et al., 2001)

Participants assigned to the exercise condition (WL+EXE) began with a 3-min warm-up at 60% HRR (HRR × 0.6 + RHR) on a bicycle ergometer. This was followed by 20 min of exercise at 80% HRR (HRR × 0.8 + RHR). This exercise intensity is categorized as vigorous according to American College of Sports Medicine guidelines (Garber et al., 2011). During the entire exercise bout, participants maintained a cadence of 75 rpm, and the resistance of the ergometer was adjusted individually to meet the target HRR. After the completion of the exercise bout, all individuals cycled at 0 W for an additional 3-min cool-down period.

### Statistical analysis

Response time in the SRTT was defined as the time from the stimulus to pressing the appropriate key associated with the visual cue. Only the response times for correct responses were included in the analysis. Furthermore, any response time longer than 2.7 SDs from a participant’s mean was removed, as was any response time exceeding 3 s (Brown et al., 2009). Skill for all TBs was determined by subtracting the average response time of the final 50 sequential trials in the block from the average response time of the 50 random trials that followed that block (Brown and Robertson, 2007a). As noted earlier, skill 1 was calculated from TB1 to determine skill at the conclusion of practice, while skill 2 was calculated from the TB2 to assess skill retention (Fig. 1). The difference (Δ) between skill 1 and skill 2 reflected the extent of procedural consolidation (i.e., off-line gains in skill performance) over the 6-h wake-filled period.

The extent of consolidation for each experimental condition (WL, VC, WL+EXE) was evaluated using a mixed-model ANOVA and targeted follow-up contrasts. A mixed-model ANOVA including the recall for the immediate and delayed (6 h) tests was also used to assess differences in declarative learning between WL and WL+EXE. Pearson’s correlations between the immediate test of the WL task and the difference between skill 1 and skill 2 in conditions WL and WL+EXE were used to explore interactions between declarative learning and procedural memory. Furthermore, to confirm that there were no associations between declarative learning and any residual explicit knowledge of the motor skill, a correlation analysis between performance on the immediate recall test of the WL task and the number of items in the SRTT sequence recalled correctly was also performed (see Brown and Robertson, 2007a).

## Results

### The interfering effects of declarative learning on procedural memory consolidation

**Figure 2.**
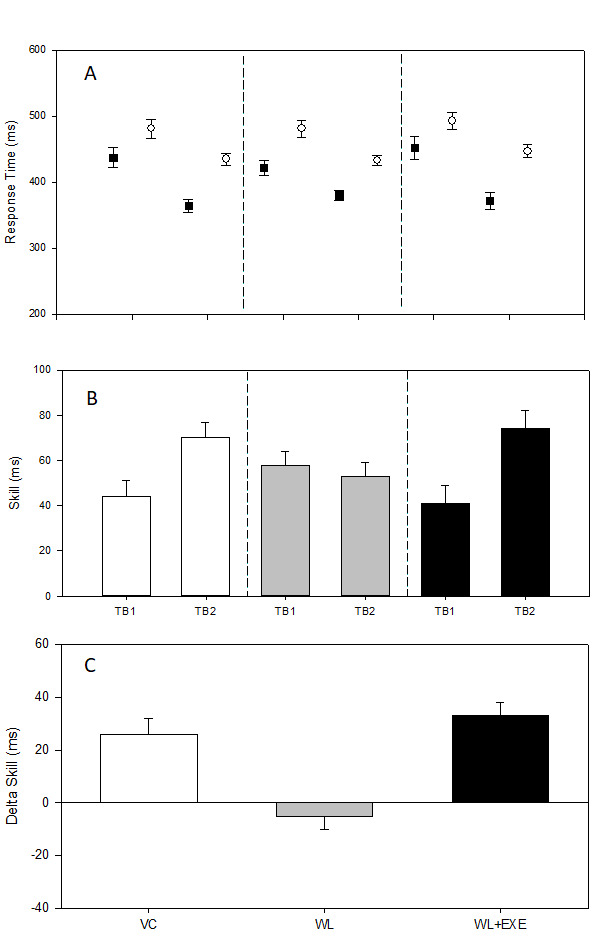
Mean response time (***A***) was calculated for the last 50 sequence trials (square symbol) and the 50 random trials (circle symbol) that occurred at the conclusion of practice of the SRTT (TB1) and again 6 h later for TB2 for individual assigned to each of the three experimental conditions (VC, WL, WL+EXE). Skill was determined as the difference between mean response time for the sequence and random trials at TB1 and again for TB2 (***B***). The difference in skill (Δ skill) between TB1 and TB2 reflects procedural consolidation and is presented for the VC, WL, and WL+EXE conditions (***C***). A larger score in this figure reflects greater procedural consolidation. These data indicate that participants in the VC and WL+EXE conditions revealed significant procedural consolidation across the 6-h wake period, which was not the case for the individuals assigned to the WL group.

Individuals assigned to the three experimental conditions (VC, WL, WL+EXE) did not differ as a function of age (*F*_(2,69)_ = 0.85, *p *=* *0.43, η_p_^2^ = 0.02), body mass index (*F*_(2,69)_ = 2.53, *p* = 0.09, η_p_^2^ = 0.07), and resting HR (*F*_(2,69)_ = 0.53, *p *=* *0.59, η_p_^2^ = 0.02; Table 1). Skill for each individual was submitted to a 3 (condition: VC, WL, WL+EXE) × 2 (TB: TB1, TB2) ANOVA with repeated measures on the last factor. This analysis revealed a significant main effect of TB, *F*_(1,69)_ = 32.67, *p *<* *0.01, η_p_^2^ = 0.32. Interpretation of the TB main effect was superseded by a significant condition × TB interaction, *F*_(2,69)_ = 13.42, *p *<* *0.01, η_p_^2^ = 0.28 (Fig. 2). Skill did not differ as a function of condition at TB1, *F*_(2,69)_ = 2.09; *p *>* *0.05, η_p_^2^ = 0.06 (VC: Mean (M)* *=* *44 ms, SEM = 7 ms; WL: M* *=* *58 ms, SEM =* *6 ms; WL+EXE: M* *=* *41 ms, SEM =* *8 ms). Targeted follow-up contrasts revealed a significant off-line gain between TB1 and TB2 when procedural learning was followed by VC, which did not involve declarative learning, *t*_(23)_ = 4.39, *p *<* *0.001 (TB1: M* *=* *44 ms, SEM =* *7 ms; TB2: M* *=* *70 ms, SEM =* *7 ms). Off-line gain was eliminated when participants experienced declarative learning following procedural skill acquisition, *t*_(22)_ = 0.93, *p *=* *0.36 (TB1: M* *=* *58 ms, SEM = 6 ms; TB2: M* *=* *53 ms, SEM =* *6 ms). Importantly, despite engaging in declarative learning, individuals exposed to a short bout of cardiovascular exercise after procedural learning but before exposure to the WL, revealed an off-line improvement, *t*_(23)_ = 6.39, *p *<* *0.001 (TB1: M* *=* *41 ms, SEM =* *8 ms; TB2: M* *=* *74 ms, SEM =* *8 ms). To verify that off-line gain after exercise was larger than off-line gain without exercise, an independent samples *t* test comparing differences in skill scores between TB1 and TB2 between WL+EXE and WL was performed. As expected, WL+EXE revealed significantly larger off-line gain compared with WL, *t*_(46)_ = 5.14, *p *<* *0.001 (WL+EX: M* *=* *33 ms, SEM =* *5 ms; WL: M = −5 ms, SEM =* *5 ms; Fig. 2*C*).

**Table 1:** Characteristics of participants included in the study

	*N*	Male	Female	Age (years; SEM)	BMI (kg/m^2^; SEM)	RHR (bpm; SEM)
VC	24	3	21	19.7 (0.3)	21.6 (0.4)	72.8 (1.0)
WL	23	9	14	20.2 (0.3)	23.3 (0.7)	71.2 (2.2)
W + EXE	25	9	16	20.3 (0.4)	22.8 (0.5)	70.8 (1.2)
Total	72	21	51	20.1 (0.20)	22.60 (0.31)	71.60 (0.86)

Number of male and females, mean age, body mass index (BMI), and resting HR (RHR) as a function of the VC, WL, and WL+EXE conditions. Data are reported as means and SEM.

These results confirmed that the introduction of a declarative learning task after the practice of the SRTT interfered with the consolidation of procedural memory, suppressing off-line gains in skill (Brown and Robertson, 2007a). More importantly, the results showed, for the first time, that the performance of a single bout of vigorous cardiovascular exercise after practicing the SRTT protects procedural memory, mitigating the interfering effects of introducing a declarative learning and returning off-line gains in skill after the consolidation period.

### The relationship between declarative learning and procedural memory consolidation

To assess differences in declarative learning between participants in the WL and WL+EXE, word recall for the immediate and delayed tests for each individual that underwent declarative learning was submitted to a 2 (condition: WL, WL+EXE) × 2 (recall test: immediate, delayed) ANOVA with repeated measures on the last factor. This analysis revealed a significant main effect of test, *F*_(1,46)_ = 16.74, *p *<* *0.01, η_p_^2^ = 0.28. This main effect was a result of poorer recall during the delayed test (WL: M* *=* *14.22 words, SEM = 0.38 words; WL+EXE: M = 14.36 words, SEM = 0.36 words) compared with that observed during the immediate test (WL: M = 14.87 words, SEM = 0.34 words; WL+EXE: M* *=* *15 words, SEM = 0.33 words) for both conditions. However, mean word recall was similar across conditions and there was no significant main effect of condition, *F*_(1,46)_ = 0.08, *p *=* *0.77, η_p_^2^ = 0.01, and condition × recall test interaction, *F*_(1,46)_ = 0.01, *p *=* *0.97, η_p_^2^ = 0.01. Taken together, these results show that there were no differences in declarative learning between WL and WL+EXE and thus that performing exercise before the WL task did not have any significant influence on WL retention (i.e., declarative learning).

On the basis of findings from Brown and Robertson (Brown and Robertson, 2007a), it was expected that the extent of an individual’s declarative learning would have a direct interfering effect on procedural memory consolidation. In other words, we hypothesized that greater WL recall would lead to smaller off-line gains in skill performance during consolidation in participants in conditions WL and WL+EXE. To evaluate this prediction, the WL recall from the immediate test for each individual in the WL and WL+EXE conditions was correlated with Δ skill (i.e., off-line gain) exhibited between TB1 and TB2. This assessment failed to reveal a significant relationship between declarative learning and off-line gains in skill performance when separate analyses were conducted for the WL (*r*^2^ = 0.007, *F *=* *0.08, *p *=* *0.79), and the WL+EXE (*r*^2^ = 0.04, *F *=* *0.39, *p* = 0.54) conditions. Moreover, combining data from the WL and WL+EXE conditions still failed to reveal a significant association between the magnitude of declarative learning and procedural consolidation (*r*^2^ = 0.01, *F *=* *0.20, *p* = 0.66).

### The relationship between declarative learning and explicit knowledge of the motor skill

A three (condition: VC, WL, WL+EXE) one-way between-subject ANOVA was conducted on the degree of explicit knowledge for the SRTT. As expected, given participants who were able to recall more than four elements in the 12-item sequence of the SRTT were excluded, this analysis failed to reveal a significant main effect of condition, *F*_(2,34)_ = 0.11, *p *=* *0.90, η_p_^2^ = 0.01. Specifically, explicit knowledge of the ordinal structure of the SRTT was similar for individuals assigned to the WL (M* *=* *2.5 elements, SEM =* *0.3 elements), VC (M* *=* *2.3 elements, SEM =* *0.3 elements), and the WL +EXE (M* *=* *2.2 elements, SEM =* *0.4 elements) conditions. Moreover, the participants’ level of declarative learning (i.e., number of words recalled during the immediate test) did not dictate the extent of explicit knowledge for the practiced SRTT exhibited by individuals in the WL (*r*^2^ = 0.01, *F *=* *0.74, *p* = 0.41) or the WL+EXE (*r*^2^ = 0.04, *F* = 2.51, *p *=* *0.14) conditions. These data indicate that there was no relationship between the declarative learning that occurred as a result of the WL task and thus that the amount of declarative learning did not influence the level of explicit knowledge of the ordinal structure of the SRTT.

## Discussion

### Exercise protects procedural memory consolidation from declarative learning interference

Memory consolidation has been described as a time-dependent process of strengthening memories typically observed as memory stabilization or off-line enhancement (McGaugh, 2000). Stabilization is most commonly described as decreased susceptibility to interference (Krakauer and Shadmehr, 2006). For example, memory for a newly acquired motor skill is reduced when the initial training used to encode this skill is followed by practice of another motor skill performed in close temporal proximity (Robertson et al., 2004). Increasing the time delay between the practice of the primary motor skill and the secondary interfering motor skill has been reported to reduce the amount of interference (Brashers-Krug et al., 1996). Given the prevailing assumption that declarative and procedural memory systems are fundamentally distinct (Squire and Zola, 1996), it is not surprising that studies investigating the protective effect of exercise on interference have focused exclusively on the declarative or procedural memory systems separately. Acute cardiovascular exercise has shown to have an enhancing effect on procedural memory during consolidation, reducing interference between motor skills acquired in close temporal proximity (Rhee et al., 2016; Lauber et al., 2017; Jo et al., 2019). The results of the present study demonstrate, that acute exercise can also protect the consolidation of procedural memory against the interfering effects of an intervening bout of declarative learning.

Two important aspects need to be considered when interpreting this novel finding. First, participants in both WL and WL+EXE conditions exhibited similar word recall for the WL task. This finding is crucial because it rules out the possibility of any potential anterograde interference effect of exercise on declarative memory, indicating that the protective effects of exercise occur through a strengthening of the procedural memory and, critically, not at the expense of exercise weakening declarative memory. Rather than selectively improving one type of memory over another, exercise appears to reduce the “bottleneck” imposed by the memory system (Breton and Robertson, 2014) thus improving the capacity to process both memories simultaneously. A second important aspect to consider refers to the fact that the period of consolidation examined during this study did not involve sleep. Previous studies have shown that when sleep is allowed, the interference effects from declarative learning on procedural memory are reduced and off-line gains in the performance of the SRTT can be obtained (Brown and Robertson, 2007a). Future studies should determine whether sleep supersedes exercise in protecting procedural memory or, alternatively, whether sleep and exercise offer unique contributions to memory consolidation (Mograss et al., 2017), thus offering potentially greater protection against the interfering effects of declarative learning.

In our study, the extent of the blockade on procedural memory consolidation was not associated with participants’ declarative learning. Our correlation analyses failed to show any significant association between off-line improvement in skill performance and the magnitude of word recall in the WL declarative task. It should be noted, however, that the level of performance in the WL task in our study was uncommonly high. A closer analysis of the data showed that 92% of the individuals from the WL and WL+EXE conditions scored at least 15 of a possible 16 words correctly while in previous studies only ∼20% of participants achieved such numbers (Brown and Robertson, 2007a). Thus, declarative learning in the present study appears to have been considerably greater and less variable across participants than what has been reported previously. The non-significant correlation between procedural and declarative learning may have resulted from the large number of individuals exhibiting a very high level of word recall. Furthermore, we did not find associations between declarative learning and the number of items identified in the SRTT. This association was not expected because we used a very stringent exclusion requirement to discard participants who relied excessively on the declarative knowledge of the sequence to acquire the SRTT (Willingham and Goedert-Eschmann, 1999). Moreover, associations between words recalled in the WL task and the number of items identified in the SRTT have been reported only when explicit versions of the SRTT have been used (Brown and Robertson, 2007b).

### Potential mechanisms underlying the protective effects of exercise on procedural memory

Acute cardiovascular exercise promotes a brain state that could optimize memory consolidation processes (Robertson and Takacs, 2017), making procedural memories more resistant to the effects of behavioral interference. Studies exploring changes in brain state potentially involved in the effects of acute exercise on procedural learning have largely focused on cortico-motor networks and, more specifically, on the primary motor cortex (M1; Singh et al., 2016). There is evidence that acute exercise increases cortico-spinal excitability (CSE; Singh and Staines, 2015), a surrogate of long-term potentiation (LTP)-like plasticity (Ziemann et al., 2004), which is essential for M1-dependent procedural learning (Rioult-Pedotti et al., 2000). Maintaining CSE after practicing the SRTT appears to be necessary for the development of off-line gains in skill performance after a wakefulness period (Tunovic et al., 2014). A single bout of cycling performed after practicing the SRTT has been reported to increase CSE assessed with transcranial magnetic stimulation (TMS) applied on the M1 representational area of the hand that performed the motor task (Ostadan et al., 2016). Increases in CSE persisted for 2 h after motor practice and were positively correlated with skill retention assessed 8 h after motor practice. Another study found that exercise reduced GABA_A_-related inhibition in M1 (Stavrinos and Coxon, 2017). When the data of the exercise and control groups were pooled together, GABA_A_ disinhibition correlated with skill retention assessed 5 h after practice. Both animal (Hess et al., 1996) and human (Ziemann et al., 2001) studies show that GABA disinhibition is needed for LTP induction in M1. Taken together, the results of these studies suggest that acute exercise can promote transient LTP-like plasticity changes in cortico-motor networks which can facilitate the creation of stronger procedural memories thus making them less susceptible to interference.

Direct mechanistic evidence of the involvement of cortico-motor networks in the protective effects of exercise on procedural memory has been provided by studies employing repetitive TMS (rTMS) protocols. rTMS can be used to modulate cortico-motor network activity and thus explore mechanisms underlying the consolidation of procedural memory (Censor and Cohen, 2011). When applied on M1, low-frequency rTMS tends to cause an inhibitory response, triggering reductions in CSE levels (Fitzgerald et al., 2006). It has been suggested that suppressing CSE after motor skill learning serves as a physiological signal that prevents subsequent motor consolidation (Tunovic et al., 2014). Muellbacher et al. (2002) were the first to apply low frequency (1 Hz) rTMS over the M1 after practicing an acceleration pinching task and showed that this inhibitory protocol cancelled the retention of the motor skill and impaired additional skill acquisition. Using the same brain stimulation paradigm, a recent study demonstrated that acute exercise can protect procedural memory from rTMS-induced interference (Beck et al., 2020). Participants practiced a visuomotor accuracy task demanding precise and fast pinch force control. Following motor practice, participants either rested or exercised for 20 min before receiving either sham rTMS or 1-Hz rTMS targeting the hand area in M1. Skill retention was evaluated 24 h following motor practice, and motor memory consolidation was operationalized as overnight changes in motor skill performance. Low frequency rTMS resulted in off-line decrements in motor performance compared with sham rTMS, but these effects were counteracted by the preceding bout of cardiovascular exercise. Since changes in CSE were not assessed, it is unclear whether the protective effects of exercise involved a preservation of CSE (Ostadan et al., 2016) against the suppressing effects of rTMS (Fitzgerald et al., 2006).

Given the crucial role of M1 on the consolidation of procedural memory (Robertson et al., 2005), the interest in this area of the brain to explain the effects of acute exercise on procedural memory is not surprising. However, it is also possible that broader network changes (Sami et al., 2014) resulting from the effects of acute exercise (Rajab et al., 2014) could also contribute to the protective effect against declarative learning induced interference demonstrated in this study. Recent studies show that the effects of acute exercise on the brain are extensive, enhancing the efficiency of functional activity and connectivity between remote cortical areas. For example, Dal Maso et al. (2018) demonstrated that a single bout of cardiovascular exercise performed immediately after motor skill learning enhanced skill retention 24 h after motor practice. The study used electroencephalography to show that exercise decreased beta band event-related desynchronization and increased functional connectivity between electrodes located over the sensorimotor areas of both hemispheres during memory consolidation. Reductions in event-related beta band desynchronization can be interpreted as an increased efficiency in usage of the neural resources to consolidate procedural memory. This increased efficiency could potentially liberate overlapping neural resources, allow the simultaneous consolidation of memories and thus reduce interference. Importantly, this study showed that skill retention was positively correlated with beta band event-related desynchronization, not only in sensorimotor motor areas, but also in prefrontal areas of the brain, including the dorso-lateral prefrontal cortex (Yanagisawa et al., 2010). This finding is relevant because acute exercise promotes transient increases in the activity of this area of the brain, which has been proposed to act as a gate, regulating the competitive interaction between procedural and declarative memories (Brown and Robertson, 2007a; Cohen and Robertson, 2011).

## Summary

The present study shows that the introduction of a single bout of exercise after practicing an implicit version of the SRTT minimizes the interfering effect of subsequent declarative learning so that procedural consolidation unfolds unhindered across a wake interval. Similar to the effects of non-invasive brain stimulation, exercise appears to have the capacity to reduce the bottleneck imposed by the brain and allow the simultaneous consolidation of procedural and declarative memories acquired in close temporal succession. An important next step will be to identify the specific neural substrates subserving the protective effects that exercise has shown to have on procedural memory against declarative learning-induced interference.

## Synthesis

Reviewing Editor: Bradley Postle, University of Wisconsin Decisions are customarily a result of the Reviewing Editor and the peer reviewers coming together and discussing their recommendations until a consensus is reached. When revisions are invited, a fact-based synthesis statement explaining their decision and outlining what is needed to prepare a revision will be listed below. The following reviewer(s) agreed to reveal their identity: Edwin Robertson, Paul Reber.

Reviewer #1

This is a very nicely written, clear, timely work that describes an interesting set of appropriately designed and analysed experiments.

The authors have quite rightly been very careful about removing from analysis those participants who developed awareness for the sequence in the motor skill memory task because this could have been sufficient to prevent/interfere with the consolidation of the task, which would otherwise have obscured the interference from the declarative learning task.

Doubtless further analysis could be conducted to look at various other aspects of learning. However, there is a danger with suggesting further analysis because this would be entirely exploratory and difficult to interpret. The work posed a question, and the current analysis provided addresses that question.

I have some comments that might improve the work further.

1. In the introduction (page 4; start of third paragraph) the authors describe competitive interactions between memory systems, and cite the work of Poldrack and Packard, 2003. I would be careful here. What that shows, and other work like it, is an interaction between different neural circuits (such as the hippocampus and striatum) - through reverse inference such work has attributed to those circuits the processing of different types of memory (declarative and motor; respectively), and through this reasoning states that there are interaction between different memory systems. However, nested within this reasoning is the flawed assumption of reverse inference - the hippocampus is not exclusively a declarative processing circuit (Schendan et al, 2003; for example) and the striatum is not exclusively involved in motor processing either.

2. The final paragraph of the introduction (page 5) gives a nice over view of the experimental design. On first reading though I got a little worried because it read as though the experimental group was the only group in which time was being inserted between the two memory tasks. Initially this seemed like a confound with two differences (exercise and time interval) between the experimental and the other groups. However, my concerns were entirely addressed by looking at Figure 1 and reading the methods, i.e., all the groups had an interval between task. As a consequence, I would be tempted to rephrase some of the sentences within that final introductory paragraph to ensure that others are not misled (or at least not able to mislead themselves!).

Reviewer #2

Summary

The report describes a finding that learning of an implicit sequential perceptuomotor task can be impeded by a subsequent explicit vocabulary learning task, and that this explicit/implicit interference can be ameliorated by a bout of cardiovascular exercise. Following 55 repetitions (15 TB1, 25 SRT ‘practice,’ 15 TB2) of a 12-item SRT sequence, an improvement in motor skill (reduction in reaction time in TB2 compared to TB1) was demonstrated following a 6-hour (awake) ‘consolidation’ period. This improvement in skill was absent in a group who additionally studied vocabulary words (explicit learning) 1 hour after SRT practice. This improvement in skill was preserved in a third group who engaged in explicit vocabulary learning, but then also engaged in a 1-hour bout of vigorous cardiovascular exercise between the implicit and explicit learning tasks. These results show that a bout of vigorous cardiovascular exercise can mitigate the interfering effects of explicit learning following motor skill acquisition.

Comments

This experiment includes a replication of a very interesting prior result (Brown & Roberston, 2007) that a verbal learning task interfering with consolidation of an implicitly learning motor sequencing skill with an equally interesting extension to a finding that this effect can be eliminated by a bout of exercise.

It is not entirely clear that all the necessary statistical details are included to support the main conclusion. The key finding of a reliable interaction is reported (line 28-30, p. 9) but because the design is complex (3x2) there need to be targeted follow-up contrasts showing that the offline gains are reliably different in the VC versus WL conditions (showing interference) and WL versus WL+EXE (showing protection). Note that the follow-ups on p. 10 (lines 5-12) showing a pattern of null and positive findings are not conclusive as they depend on a weaker null-result finding for the WL condition. It looks like these analyses would be reliable, so the lack of inclusion is probably just an oversight.

The one other minor interpretation concern would be that the verbal report to exclude explicit sequence learning is probably not the most sensitive measure. Recognition measures (e.g., Willingham, Greely & Bardone, 1993) appear to be the most sensitive way to detect explicit sequence knowledge in healthy participants. However, the results are interesting enough to stand even if the possibility of some contribution of explicit sequence knowledge cannot be completely ruled out.

The manuscript is not a model of clarity in writing. The methods, results and conclusions are all quite difficult to unpack and undercut the efficacy of communicating the interesting findings.

The speculation about mechanisms in the conclusion is necessarily speculative. It’s not immediately clear what kind of neural mechanism would account for the SRTT and WL task interaction since these tasks are associated with different brain systems (SRTT most closely associated with BG function, WL with MTL; n.b. the idea that the MTL is involved in SRTT has been suggested in some neuroimaging studies with healthy participants but these are necessarily non-causal). However, the results are surprising and interesting enough to report even if a definitive mechanistic conclusion is not immediately obvious.
